# Associations between Resident Perceptions of the Local Residential Environment and Metabolic Syndrome

**DOI:** 10.1155/2012/589409

**Published:** 2012-09-25

**Authors:** Katherine Baldock, Catherine Paquet, Natasha Howard, Neil Coffee, Graeme Hugo, Anne Taylor, Robert Adams, Mark Daniel

**Affiliations:** ^1^Social Epidemiology and Evaluation Research Group, Sansom Institute for Health Research and School of Health Sciences, University of South Australia, Adelaide, SA 5001, Australia; ^2^Research Centre, Douglas Mental Health University Institute, Montreal, QC, Canada H4H 1R2; ^3^Discipline of Geography, Environment and Population, The University of Adelaide, Adelaide, SA 5000, Australia; ^4^Population Research and Outcome Studies, Discipline of Medicine, The University of Adelaide, Adelaide, SA 5000, Australia; ^5^The Health Observatory, The Queen Elizabeth Hospital Campus, The University of Adelaide, Adelaide, SA 5011, Australia; ^6^Department of Medicine, St Vincent's Hospital, The University of Melbourne, Fitzroy, VIC 3065, Australia

## Abstract

A substantial body of research has arisen concerning the relationships between objective residential area features, particularly area-level socioeconomic status and cardiometabolic outcomes. Little research has explored residents' perceptions of such features and how these might relate to cardiometabolic outcomes. Perceptions of environments are influenced by individual and societal factors, and may not correspond to objective reality. Understanding relations between environmental perceptions and health is important for the development of environment interventions. This study evaluated associations between perceptions of local built and social environmental attributes and metabolic syndrome, and tested whether walking behaviour mediated these associations. Individual-level data were drawn from a population-based biomedical cohort study of adults in Adelaide, South Australia (North West Adelaide Health Study). Participants' local-area perceptions were analysed in cross-sectional associations with metabolic syndrome using multilevel regression models (*n* = 1, 324). A nonparametric bootstrapping procedure evaluated whether walking mediated these associations. Metabolic syndrome was negatively associated with greater *local land-use mix*, positive *aesthetics*, and greater *infrastructure for walking*, and was positively associated with greater perceived *crime* and *barriers to walking*. Walking partially mediated associations between metabolic syndrome and perceived environmental features. Initiatives targeting residents' perceptions of local areas may enhance the utility of environmental interventions to improve population health.

## 1. Introduction

The rising prevalence of cardiometabolic diseases, including type 2 diabetes and cardiovascular disease, presents a major international public health challenge for the 21st century [1]. Public health prevention efforts to combat these diseases have largely focused on identifying and reducing individual-level risk factors, particularly physical inactivity and poor nutrition. Such prevention strategies alone, however, have had poor utility against increasing morbidity and mortality attributable to cardiometabolic diseases at the population level [2, 3]. There is a growing recognition that strategies which target individual behaviours in addition to the context within which such behaviours arise can have the greatest public health impact [4]. This has led to a rapidly increasing body of research on the role of residential, or local-area, environments in patterning cardiometabolic risk factors and subsequent disease. 

Consistent associations have been established between measures of area socioeconomic status and cardiometabolic diseases [5–7]. Fewer studies have examined other area features, such as road traffic [8], population density [9], or neighbourhood social cohesion [10]. A growing body of evidence demonstrates relations between resident perceptions of local area features and risk factors for cardiometabolic diseases, particularly physical activity and obesity. Body mass index and physical activity are associated with perceived environmental features often indicative of more “walkable” areas, such as greater land-use mix [11–13], infrastructure for walking [12, 14, 15], positive aesthetics [11–13, 15–19], and safety from crime [19–25]. Limited research, however, has examined perceived environmental features in relation to a measured cardiometabolic outcome. 

Recent research makes clear the public health importance of understanding and targeting resident perceptions of their local area [26, 27]. However, although perceptions of environmental features are frequently used to represent the objective reality [28], several studies have shown a general lack of correspondence between perceived and objective measures of environments [29, 30]. In order to better understand perceptions of environments as they relate to health outcomes, it is essential to improve the conceptualisation of perceptual measures.

Perceptions of environmental features reflect an individual's interaction with a particular environment, involving both *perception*, with inputs from the visual, haptic, auditory, and other senses [31], and *proprioception*, the experience of one's own body in space [32]. These multisensory inputs are integrated to form a cognitive representation of the environment. The understanding and meaning subsequently attached to these cognitive representations are influenced by factors such as language, social class, personal values, place attachment, culture, social norms, past experiences, physical capacity, and individual personality characteristics [29, 33, 34]. Perceptions of environments thus constitute a mix of individual and broader societal factors, and are not simply proxy measures for specific objective environmental features.

Blacksher and Lovasi [27] have argued that inadequate attention has been given to people's understandings of their neighbourhood in developing built environment interventions. They suggest that the primary supposition in much place and health research is that changes to the built environment will lead to positive changes in behaviour for those within the changed environment, thereby resulting in better health. Yet, a limited understanding of the relationship between environmental perceptions and health outcomes, and the pathways linking perceptions to health, may yield ineffective built environment interventions aimed at improving health. For example, the availability or accessibility of environmental features presumed to be important for health may have little effect on health outcomes if they are not perceived to be available or accessible [35]. 

In addition to the need for evaluating relations between perceived environmental attributes and cardiometabolic outcomes, it is important to explore the mechanisms that may underpin such relations. It has been proposed that environmental perceptions may predict cardiometabolic health indirectly through behaviours such as physical activity [36], adverse emotional and affective states, and chronic stress [37, 38], and directly through harmful psychological and physiological responses that contribute to the development of obesity and cardiometabolic diseases [37, 39, 40]. Few studies, however, have formally tested the pathways of such associations; for example, whether physical activity mediates the associations between perceived environmental features and health outcomes [41]. Information regarding explanatory mechanisms is essential, however, to improving our understanding of causal processes. 

This study aimed to evaluate whether perceived features of local residential areas were associated with a clinically measured cardiometabolic outcome, namely, metabolic syndrome. Metabolic syndrome is a clustering of clinical risk factors strongly predictive of cardiometabolic diseases [42–44]. It has utility as a metric for evaluating area-level relationships between environmental factors and population-level risk for cardiometabolic outcomes [37, 45]. A secondary aim was to assess whether associations between perceived environmental features and metabolic syndrome were mediated by walking behaviour. 

## 2. Methods

### 2.1. Study Sample

This cross-sectional investigation used data from the North West Adelaide Health Study (NWAHS) conducted in Adelaide, Australia. The NWAHS is a longitudinal representative cohort of 4,056 randomly selected adults aged 18 years and over, originally recruited between 2000 and 2003 from the northern and western metropolitan regions of Adelaide [46, 47]. In 2001, the north west region comprised 38% of the Adelaide metropolitan population, and 28% of the South Australian population [48]. Three waves of data collection for the NWAHS have been undertaken to date. NWAHS data collected across Wave 2 (2004–2007) were utilised for this cross-sectional analysis, this being the only period for which all required measures were available. Information on self-reported sociodemographic variables and health behaviours were collected via telephone interview. Participants attended a clinic where biomedical measurements were taken. Information on current medications prescribed for participants was obtained by linking Australian Pharmaceutical Benefits Scheme (PBS) data to each individual participant using their Medicare number. Perceptions of local residential area features were obtained via postal or online questionnaire. All participants with a valid residential address were assigned a georeference corresponding to their place of residence at the time of the Wave 2 clinic visit. Socioeconomic measures pertaining to residential areas were represented at the State Suburb level. Suburbs are formed by aggregating Census Collection Districts [49]. Relative to alternate area-level units, the Suburb provides strong between-unit variability while avoiding the problem of small cluster sizes associated with using smaller spatial units such as Census Collection Districts. This study was approved by the Ethics of Human Research Committees of the Central Northern Adelaide Health Service, the University of South Australia, and the South Australian Department of Health.

### 2.2. Measures

#### 2.2.1. Outcome Measure

Metabolic syndrome was classified using the International Diabetes Federation criteria [50], including central obesity (defined as waist circumference ≥94 cm for Europid men and ≥90 cm for non-Europid men, and ≥80 cm for Europid and non-Europid women), plus any two of the following four factors: raised triglyceride level (>1.7 mmol/L); reduced HDL cholesterol (<1.03 mmol/L in males and <1.29 mmol/L in females), or treatment for lipid abnormality; raised blood pressure (systolic blood pressure ≥130 or diastolic blood pressure ≥85 mm Hg), or treatment for hypertension; raised fasting plasma glucose (FPG; ≥5.6 mmol/L), or previously diagnosed type 2 diabetes. Biomedical examinations for obtaining waist circumference, triglyceride, HDL cholesterol, blood pressure and FPG measurements were undertaken at one of two hospital-based clinics. A structured protocol was followed by trained clinic staff conducting the biomedical examinations. A fasting blood sample of approximately 30 mL was taken as part of the biomedical examination for obtaining triglyceride, HDL cholesterol, and FPG levels. Blood pressure was measured using a standard, calibrated sphygmomanometer. The average of two readings, taken five to ten minutes apart while the participant was seated and relaxed, was used. Waist circumference was measured to the nearest 0.1 centimetre using an inelastic tape maintained in a horizontal plane, with the subject standing comfortably with weight distributed evenly on both feet. The measurement was taken at the level of the narrowest part of the waist. Physician-diagnosed type 2 diabetes was reported by respondents via telephone interview and written questionnaire. International Diabetes Federation metabolic syndrome criteria for dyslipidaemia or hypertension were considered met if a participant had been prescribed medication to treat such conditions in the six months prior to their Wave 2 clinic attendance.

#### 2.2.2. Perceptions of the Local Residential Environment

Perceptions of local-area features were assessed using six subscales of the Australian version of the Neighbourhood Environment Walkability Scale (NEWS-AU) [51], a modified version of the NEWS [52]. This scale captures several dimensions of the local built and social environmental context which could influence cardiometabolic outcomes through walking behaviour. The items from the following six subscales were used in this analysis: *land-use mix—diversity*, *access to services*, *infrastructure for walking/cycling*, *aesthetics*, *traffic safety*, and *crime safety*. All items were rated on a four-point Likert scale, except *land-use mix—diversity*. The *land-use mix—diversity* subscale assesses perceived walking proximity to 24 types of stores and resources (e.g., supermarket, library, or park), for which the scorable walking distance ranged across five categories from 1–5 minutes to more than 30 minutes. Resources included in the *land-use mix—diversity* subscale were defined for this analysis as being locally available if perceived to be within a 20-minute walking distance from home (approximately equivalent to 1.6 kilometres or 1 mile) [53, 54]. The number of different types of locally available resources was summed to give a score out of 24, indicating *local land-use mix*. This score was then standardised to give a mean of zero and a standard deviation of 1. 

Recently, Cerin and colleagues [55] recommended a modified scoring procedure for the NEWS-AU, based on the results of a factor analysis of all subscales except *land-use mix—diversity*. This subscale was excluded from the factor analysis due to the nature of the scale and response format. In our sample, application of this NEWS-AU scoring procedure resulted in a modest internal consistency (Cronbach's alpha) for certain subscales: *infrastructure for walking/cycling* = 0.64, *traffic load* = 0.68, and *traffic safety* = 0.45. The other subscales demonstrated acceptable internal consistency: *access to services* = 0.81, *aesthetics* = 0.77, and *crime* = 0.79. To improve the internal consistency of the perceived environment measures and find the most appropriate item structure for our sample, an exploratory factor analysis was undertaken with the items rated on a four-point scale from the NEWS-AU [51] subscales *access to services*, *infrastructure for walking/cycling*, *aesthetics*, *traffic safety*, and *crime safety*. Following the rationale of Cerin and colleagues [56], the *land-use mix—diversity *subscale was excluded from the factor analysis. The standardised factor scores from this exploratory factor analysis were retained and used in subsequent analyses to represent perceived environmental features, along with the standardised *local land-use mix* score. Higher values for the *local land-use mix*, *aesthetics*, *infrastructure for walking*, and *access to services* measures indicate more positive perceptions of those area features. Higher scores on the *crime* and *barriers to walking* factors indicate more negative perceptions of those area features.

#### 2.2.3. Mediator

Walking behaviour was measured via a single item in a self-report questionnaire, which asked respondents to report the total amount of time (in hours and minutes) they had spent walking for sport, recreation or fitness in the previous two weeks. The survey item was replicated from the Australian National Health Surveys undertaken in 2001 and 2004 [57], and has demonstrated acceptable test-retest reliability [58]. Walking behaviour in this study was expressed as the average time spent walking over one week. 

#### 2.2.4. Covariates

Self-reported socio-demographic characteristics included: age, sex, marital status assessed as married/living with partner or not partnered, annual household income assessed as $20,000 or less, $20,001 to $60,000, or greater than $60,000, work status assessed as employed or not employed, and educational attainment assessed as less than Bachelor's degree, or Bachelor's degree or higher. There were *n* = 10 (0.8%) missing data for educational attainment and *n* = 34 (2.6%) missing data for household income at Wave 2 of the NWAHS. Missing values for these variables were replaced by Wave 1 values, to avoid loss of Wave 2 observations. Median weekly household income was utilised as a measure of area-level socioeconomic status. This measure, extracted at the suburb level from the 2006 Australian Bureau of Statistics Census of Population and Housing [59], was ascribed to each participant. 

### 2.3. Data Analysis

#### 2.3.1. Exploratory Factor Analysis of NEWS-AU Items

An exploratory factor analysis was undertaken in SPSS (version 18.0, SPSS Inc., Chicago, IL, USA) for participants with complete data on all items to be factor analysed. The principal components method with an oblique rotation was used to allow for a correlated factor structure. Eigenvalues and scree plots were examined for all solutions. Factor structures were compared with the original NEWS, the NEWS-AU, and the respecified NEWS-AU structures. Several factor extractions were attempted in order to achieve a parsimonious model conceptually comparable to the original scales. 

#### 2.3.2. Evaluation of Associations between Perceived Environmental Features, Walking Time, and Metabolic Syndrome

**Figure 1:**
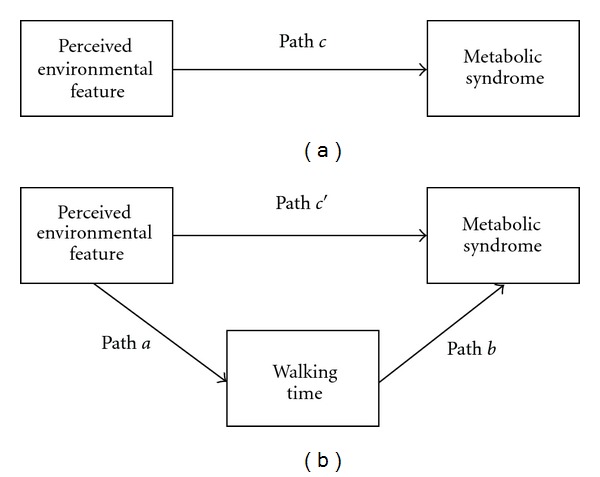
(a) Direct association between perceived environmental features and metabolic syndrome. (b) Indirect association between perceived environmental features and metabolic syndrome through walking time.

Associations between perceived environmental features, walking time, and metabolic syndrome were assessed for participants with data for all measures using a series of multilevel regression models that accounted for the clustering of individuals within suburbs. Individuals were modelled at the first level, and suburbs at the second level, using the SAS (version 9.1.3; SAS Institute Inc., Cary, NC, USA) glimmix procedure. The mediating role of walking time in associations between environmental perceptions and metabolic syndrome was evaluated using the criteria of Baron and Kenny [60]. Figures 1(a) and 1(b) present the paths to be tested under these criteria. All analyses included participant age, sex, marital status, income, education, work status, and area-level income; subsequent models also included walking time. Direct associations between each perceived environmental feature and metabolic syndrome (Path *c*), and indirect associations between each perceived environmental feature and metabolic syndrome, accounting for walking time (Path *c*′), were estimated in separate models using multilevel logistic regression. To formally test the mediating effect of walking time, associations between each perceived environmental feature and walking time (Path *a*) were estimated using multilevel Poisson regression, and associations between walking time and metabolic syndrome, controlling for each perceived environmental feature (Path *b*), were estimated in separate multilevel logistic regression models. Mediated (indirect) effects were then formally tested using a nonparametric bootstrapping procedure (*n* = 20,000 samples) [61] which estimates the sampling distribution of the indirect effect (*ab*) and the corresponding 95% confidence interval (CI). We used this method because it is one of the more valid and powerful methods for testing intervening, or mediating, variable effects, particularly where the indirect effect is not normally distributed, and for multilevel models with binary outcomes [62]. Indirect effects were considered significant when the 95% CI did not include zero. For all tests, statistical significance was considered at *P* = 0.05. 

## 3. Results

A total of *n* = 1,656 individuals provided complete responses to questions on perceptions of *access to services*, *infrastructure for walking/cycling*, *aesthetics*, *traffic safety*, and c*rime safety *in the local area. Data from these participants were used in the factor analysis of NEWS-AU items. Of these 1,656 participants, those with missing information for the *local land-use mix *subscale, clinical measurements, demographic information, and self-reported walking time (*n* = 320), and those without a valid residential address who could not be ascribed an area-level income value (*n* = 12) were excluded from subsequent analyses.  Table 1 presents the demographic characteristics of the final analytic sample (*n* = 1, 324). Participants in this sample were clustered within 201 Suburbs, with a median of three participants per Suburb (interquartile range = 8). The prevalence of metabolic syndrome in this sample was 35.2% (95% CI 32.7, 37.8). 

### 3.1. Exploratory Factor Analysis of NEWS-AU Items

Initial factor analysis solutions for the 31 NEWS-AU items analysed yielded either too many or too few factors for meaningful interpretation, based on the eigenvalues > 1 criterion and scree plot. In order to compare more directly with the NEWS and two NEWS-AU structures, we forced a six- and a five-factor solution. The six-factor solution was very similar to the re-specified NEWS-AU structure determined for this scale by Cerin et al. [55], but the derived factors in our sample did not all demonstrate acceptable reliability as determined by Cronbach's alpha. A five-factor solution was selected for further analysis due to its parsimony, reliability, and similarity to the original NEWS subscales. The five correlated factors accounted for 45.6% of the total variance (Table 2), and represented *aesthetics*, *crime*, *infrastructure for walking*, *access to services* and *barriers to walking *in the local area. The factor *infrastructure for walking* was reverse scored to be interpreted in the same direction as the original items included in the factor. The factor structure, including item loadings, is presented in Table 3. 

### 3.2. Associations between Environmental Perceptions and Metabolic Syndrome, and the Mediating Effect of Walking Behaviour (Paths *c* and *c*′)

All but one of the perceived environmental features were associated with the presence of metabolic syndrome, in models accounting for participant age, sex, marital status, income, education, work status, and area-level income (Path *c*, Table 4). Attributes of the perceived environment that were negatively associated with metabolic syndrome included *local land-use mix*, *aesthetics*, and *infrastructure for walking* in the local area. Perceived environmental features positively associated with metabolic syndrome included *crime* and *barriers to walking* in the local area. *Access to services* was not associated with having metabolic syndrome. With the inclusion of walking time, the effect of perceived environmental features on metabolic syndrome generally remained unchanged (Path *c*′, Table 4) indicating limited mediation by walking behaviour.

### 3.3. Associations between Environmental Perceptions and Walking Time (Path a)

All but one of the perceived environmental attributes were associated with walking time in the expected direction, accounting for covariates. Perceived environmental features positively related to walking included: *local land-use mix* (estimate = 0.08 (95% CI 0.08, 0.09), *P* < 0.0001), *aesthetics *(estimate = 0.06 (95% CI 0.06, 0.07), *P* < 0.0001), and *access to services *(estimate = 0.17 (95% CI 0.16, 0.18), *P* < 0.0001). Perceived environmental features negatively related to walking included: *crime* (estimate = −0.10 (95% CI −0.11, −0.10), *P* < 0.0001) and *barriers to walking *(estimate = −0.03 (95% CI −0.04, −0.03), *P* < 0.0001). The *infrastructure for walking *factor was associated with walking, but not in the expected direction (estimate = −0.02 (95% CI −0.03, −0.01), *P* < 0.0001).

### 3.4. Associations between Walking Time and Metabolic Syndrome, Adjusting for Environmental Perceptions (Path b)

The number of minutes walked over one week was associated with metabolic syndrome, while adjusting for covariates and each perceived environmental feature. In all models accounting for covariates and each individual perceived environmental feature, walking time was negatively associated with having metabolic syndrome (OR = 0.97 (95% CI 0.95, 0.99), *P* < = 0.005).

### 3.5. Indirect Effect of Walking Time in Associations between Environmental Perceptions and Metabolic Syndrome

The indirect effect of walking was formally tested where both path *a* (association between each perceived environment feature and walking time) and path *b* (association between walking time and metabolic syndrome) were significant. Estimates of the indirect effect of walking time are presented in Table 5. Walking time mediated associations between metabolic syndrome and *local land-use mix*, *aesthetics*, *crime*, *access to services* and *barriers to walking*. These indirect effects were small, however none of the 95% CIs encompassed zero. The indirect effect of walking was strongest in associations between metabolic syndrome and perceived *access to services* and *crime*. 

## 4. Discussion

This cross-sectional study of adults drawn from a metropolitan area demonstrated consistent relationships between residents' perceptions of local-area attributes and metabolic syndrome. Walking behaviour was a weak, partial mediator of these associations.

Previous research investigating relations between neighbourhoods and cardiometabolic diseases has largely represented local residential areas using measures of area-level socioeconomic status. These studies have demonstrated associations between area-level socioeconomic deprivation and ischaemic heart disease mortality [63], coronary heart disease mortality [64, 65], coronary heart disease incidence [5, 6, 66], coronary heart disease risk factors [67], type 2 diabetes incidence [7], and components of the insulin resistance syndrome [68]. Other studies have found that individual socioeconomic factors largely accounted for associations between area deprivation and coronary heart disease [69–72]. 

Research investigating cardiometabolic diseases in relation to features of environments other than socioeconomic status has evidenced a variety of associations. For instance, ischaemic heart disease is associated with population density [9] and neighbourhood residential stability [73]. Coronary heart disease has been linked to neighbourhood crime [74, 75], exposure to road traffic [8, 76], and neighbourhood social capital [77]. Insulin resistance is associated with distance to wealthy areas [78], and acute myocardial infarction mortality is similarly associated with neighbourhood social cohesion and perceived safety [10]. The majority of these studies tended to examine only one measure of the residential environment other than area-level socioeconomic status in relation to cardiometabolic health outcomes. The present study extends this body of research in demonstrating associations between a number of specific perceived features of the built and social environment and a clinically measured cardiometabolic outcome. 

Knowledge of perceived environmental features that are related to cardiometabolic outcomes is relevant to the development of environmental interventions aimed at improving health. Changes in the environment may also require educational interventions targeting residents' perceptions of the environment. For example, the introduction of large scale food retailing in a disadvantaged community in the United Kingdom had little effect in improving diet and health [79]. A study from the United States similarly found that local availability of supermarkets and grocery stores was not related to diet quality [80]. It may be that use of local fresh food stores is related to residents' perceptions of food availability and affordability which could be improved through more targeted interventions, for example campaigns aimed at food awareness, affordability, and acceptability [35]. 

In addition to identifying the perceived features of residential environments associated with metabolic syndrome, efforts to elucidate the mechanisms underlying these associations can aid in understanding causal processes. Such knowledge is important for supporting the development of public health interventions [81]. Studies exploring physical activity as a mediator that links perceptions of various local-area attributes to cardiometabolic risk have shown mixed results. Some studies have presented results suggestive of mediation by physical activity [41, 82, 83]. Other studies have not found evidence for mediation by physical activity [10, 84]. Research linking local-area socioeconomic indicators to cardiometabolic health through physical activity has also had mixed findings. Some studies have indicated partial mediation by physical activity [85, 86], while others have not [13, 14]. Only two studies have assessed physical activity as a mediator independent of other risk factors for cardiometabolic disease [41, 84]. None of the other studies reviewed here [10, 13, 14, 82, 83, 85, 86] could therefore determine the specific effect of physical activity as a mediator. Just one study thus far published has formally tested the statistical significance of the mediated effect of physical activity [41].

In our sample, walking behaviour was a weak, though statistically significant mediator linking a range of perceived features of the local residential environment to metabolic syndrome. The stronger indirect effect of walking in associations between metabolic syndrome and perceived *access to services* and *crime *may have been influenced by the stronger associations between these two perceived environmental features and walking behaviour, compared to other environmental features. The indirect effect of walking was smaller for the other perceived environmental features, and generally weak for all perceived environmental features; however, other potential untested mediators such as chronic stress [37, 38] may be operating in these associations. 

An unexpected finding in this study was that positive perception of infrastructure for walking was inversely related to walking time. It is possible that the perceived presence of infrastructure for walking alone may not be a sufficient support for greater walking. For example, greater walking may require positive infrastructure as well as perceived access to a range of destinations or services. Alternatively it is possible that a third, unmeasured variable related to both perceptions of *infrastructure for walking* and walking behaviour could explain this result (i.e., residual confounding). Due to the cross-sectional nature of this study, it is also possible that reverse causality is in effect: that reduced walking behaviour means that residents are not aware of the problematic or unsupportive infrastructure for walking.

Comprehensive measurement of the perceived local residential environment, clinically measured metabolic syndrome, and formal mediation analysis in a large population-based sample are the main strengths of this study. Limitations of this study include the fact that analyses were based on cross-sectional data. Hence, while the direction of associations can be hypothesised, these remain to be properly assessed using longitudinal data. Other potential mediating factors, not tested in the present study due to lack of suitable measures, could also influence environment-health relationships, including diet and chronic stress.

Our data indicate that perceived measures of the local residential environment are related to metabolic syndrome. Walking behaviour was a weak statistical mediator of this association. Whether this weak association reflects the characteristics of our measure, or other untested mediators including psychosocial factors and chronic stress, remains to be determined. These findings suggest that public health and urban planning strategies aimed at improving population health by changing environments might also consider targeting residents' perceptions of area features, as a means to enhance the effectiveness of such interventions. 

## Figures and Tables

**Table 1:** Demographic characteristics of the sample (*n* = 1, 324).

	*n*	Mean (SD) or % (95% CI)
Age (years)	1324	54.3 (14.3)
Sex		
Male (%)	609	46.0 (43.3–48.7)
Female (%)	715	54.0 (51.3–56.7)
Marital status		
Married, living with partner (%)	916	69.2 (66.7–71.6)
Separated, divorced, widowed, never married (%)	408	30.8 (28.3–33.3)
Education level		
Less than Bachelor degree (%)	1139	86.0 (84.1–87.8)
Bachelor degree or higher (%)	185	14.0 (12.2–15.9)
Annual household income		
Less than $20,001 (%)	298	22.5 (20.3–24.8)
$20,001 to $60,000 (%)	633	47.8 (45.1–50.5)
More than $60,000 (%)	393	29.7 (27.3–32.2)
Work status		
Employed (%)	735	55.5 (52.8–58.2)
Not employed (%)	589	44.5 (41.8–47.2)
Area-level median weekly household income (AUD)	1324	864.05 (201.36)
Walking time in previous week (mins)	1324	113.4 (196.8)

**Table 2:** Characteristics of factors derived from the Australian version of the Neighbourhood Environment Walkability Scale (*n* = 1, 656).

Factor	No. of items	Percent of variance explained	Cronbach's alpha^a^
Aesthetics	6	17.75	0.73
Crime	6	11.78	0.80
Infrastructure for walking	10	6.05	0.74
Access to services	3	5.20	0.85
Barriers to walking	6	4.79	0.58

^a^Based on items with loadings ≥ 0.4.

**Table 3:** Factor structure of the Australian version of the Neighbourhood Environment Walkability Scale (*n* = 1, 656).

Item no.^a^	Item	Item loading on each factor^b^				
		Factor 1	Factor 2	Factor 3	Factor 4	Factor 5
A1	Can do most shopping				0.81	
A2	Many shops within easy walking distance				0.89	
A3	Many places to go within easy walking distance				0.85	
A4	Easy to walk to public transport stop			−0.54		
A5	Streets in local area are hilly			0.39		
A6	Major barriers to walking					0.53
A7	Car parking difficult in shopping areas					0.50
B1	Footpaths on most of the streets			−0.75		
B2	Footpaths are well maintained			−0.63		
B3	Park or nature reserve easily accessible			−0.43		
B4	Grass/dirt strip separating streets from footpaths			−0.58		
B5	Footpaths separated from road/traffic by parked cars					0.28
B6	Bicycle or walking paths easily accessible			−0.50		
C1	Lots of greenery around the local area	0.63				
C2	Tree cover or canopy along footpaths	0.50				
C3	Many interesting things to look at	0.74				
C4	Local area free from litter, rubbish, or graffiti		0.53			
C5	Attractive buildings and homes	0.67				
C6	Pleasant natural features	0.70				
D1	Lots of traffic along most nearby streets					0.68
D2	Live on or near main arterial road or throughway for motor vehicles					0.58
D3	Speed of traffic usually slow	0.29				
D4	Many traffic slowing devices			−0.38		
D5	Busy streets have pedestrian crossings and traffic signals			−0.50		
D6	A lot of exhaust fumes					0.61
E1	Streets are well lit at night			−0.51		
E2	A lot of petty crime		0.78			
E3	A lot of major crime		0.79			
E4	Level of crime makes it unsafe to walk during the day		0.66			
E5	Level of crime makes it unsafe to walk at night		0.82			
E6	Feel safe walking home from a bus or train stop at night		−0.62			

^a^NEWS-AU subscales: [51] A: access to services; B: infrastructure for walking/cycling; C: aesthetics; D: traffic safety; E: crime safety. ^b^Factors derived from this analysis: factor 1: aesthetics; factor 2: crime; factor 3: infrastructure for walking; factor 4: access to services; factor 5: barriers to walking.

**Table 4:** Multivariable associations between each feature of the perceived environment and metabolic syndrome (*n* = 1, 324).

	Model 1, Path c^a^			Model 2, Path c^′b^		
	Odds ratio	95% CI	*P* value	Odds ratio	95% CI	*P* value
Local land-use mix	0.87	0.77, 1.00	0.04	0.87	0.77, 1.00	0.04
Aesthetics	0.88	0.77, 1.00	0.04	0.88	0.78, 1.00	0.06
Crime	1.15	1.01, 1.31	0.04	1.15	1.01, 1.31	0.04
Infrastructure for walking	0.85	0.75, 0.97	0.01	0.85	0.75, 0.97	0.02
Access to services	0.93	0.82, 1.05	0.24	0.95	0.84, 1.07	0.39
Barriers to walking	1.16	1.03, 1.32	0.02	1.16	1.02, 1.31	0.02

^a^Adjusted for participant age, sex, marital status, income, education, work status, and area-level income. ^b^Adjusted for participant age, sex, marital status, income, education, work status, area-level income, and walking time.

**Table 5:** Indirect effect of walking time in associations between perceived environmental features and metabolic syndrome (*n* = 1, 324).

Perceived environmental feature	Indirect effect estimate (*ab*)	95% CI
Local land-use mix	−0.00253	−0.00428, − 0.000692
Aesthetics	−0.00196	−0.00337, − 0.000595
Crime	0.00314	0.000960, 0.00538
Access to services	−0.00530	−0.00906, − 0.00161
Barriers to walking	0.00097	0.000307, 0.00174
